# 2194. Implementation of the CDC’s Core Elements of Antibiotic Stewardship: A Survey of Long-Term Care Facilities During the COVID-19 Pandemic

**DOI:** 10.1093/ofid/ofad500.1816

**Published:** 2023-11-27

**Authors:** Catherine (Cathy) Cichon, Jenna Preusker, Mounica Soma, Danny Schroeder, Trevor C Van Schooneveld, Scott J Bergman, Muhammad Salman Ashraf

**Affiliations:** UNMC, Omaha, Nebraska; Nebraska Medicine/Nebraska DHHS, Norfolk, Nebraska; Nebraska Medicine, Omaha, Nebraska; Nebraska Medicine, Omaha, Nebraska; University of Nebraska Medical Center, Omaha, NE; Nebraska Medicine, Omaha, Nebraska; University of Nebraska Medical Center, Omaha, NE

## Abstract

**Background:**

Nebraska Antimicrobial Stewardship Assessment and Promotion Program (ASAP) is funded by the Nebraska DHHS to assist healthcare facilities with improving their antimicrobial stewardship programs (ASP). ASAP conducted a survey during the COVID-19 pandemic to learn about implementation of the CDC core elements (CE) of antibiotic stewardship in long-term care facilities (LTCF).

**Methods:**

An online self-assessment survey of CE was created and hosted on the ASAP website. A link to the survey was shared with LTCF through ASAP’s email distribution lists, webinars, and other meetings. Data were summarized using descriptive statistics.

**Results:**

54 of 196 licensed LTCF in Nebraska responded to the survey between 05/2021 to 12/2022. Of these, 26 (48%) had all 7 CE with reporting and education being the least likely CE to be met (Tables 1 and 2). Half (50%) of LTCF did not have a signed leadership statement of support for ASP. ASP leadership responsibility was often shared across multiple positions (physicians, directors or assistant directors of nursing [DON/ADON], pharmacists, and infection preventionists), but DON/ADON were most commonly accountable (69%). Consultant pharmacists were the most commonly identified ASP experts (74%) available to LTCF. Only 57% of LTCF reported successfully implementing at least one infection-specific intervention to improve antibiotic use (AU); addressing UTIs was the most commonly identified intervention (31%). Almost a quarter (22%) of LTCF did not monitor rates of *C. difficile* infection, and about a third (30%) did not track antibiotic-resistant (AR) organisms. While 78% of LTCF provided educational resources to their nursing staff on AR and opportunities to improve AU, only 48% provided these resources to their clinical providers. Out of 26 LTCF reporting areas of antibiotic misuse at their facility, 88% agreed that ASP could help address the issue. For 24 LTCF that reported barriers to starting or improving ASP, prescriber resistance and lack of time were the most commonly identified barriers (28% and 13%).

**Figure:**
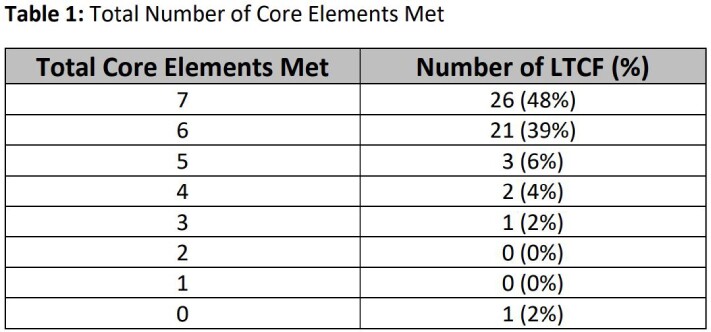

**Figure:**
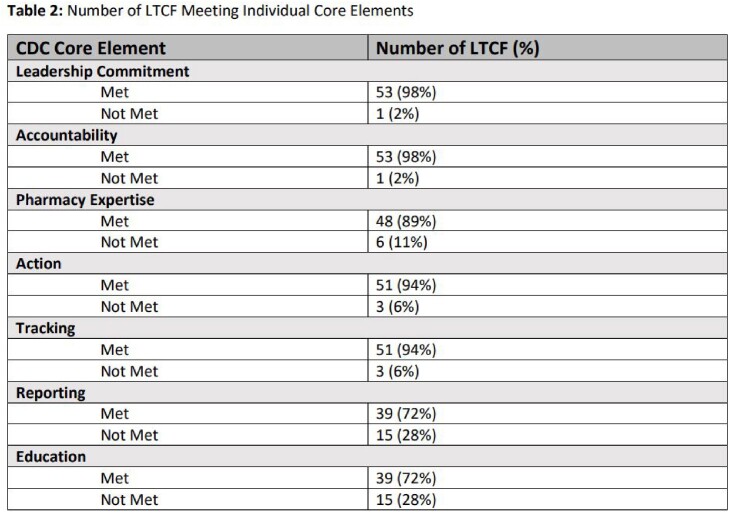

**Conclusion:**

Implementation of the CE remains low in LTCF and was likely further impacted by the COVID-19 pandemic. The findings highlight the challenges to implementing ASP in LTCF along with opportunities for improvement.

**Disclosures:**

**Trevor C. Van Schooneveld, MD, FSHEA, FACP**, AN2 Therapeutics: Grant/Research Support|Biomeriuex: Advisor/Consultant|Biomeriuex: Grant/Research Support|Insmed: Grant/Research Support|Thermo-Fischer: Honoraria **Scott J. Bergman, PharmD**, bioMerieux, Inc.: Honoraria **Muhammad Salman Ashraf, MBBS**, Merck & Co. Inc: Board Member|Merck & Co. Inc: Grant/Research Support

